# Different Effects of Aging on Intraocular Pressures Measured by Three Different Tonometers

**DOI:** 10.3390/jcm10184202

**Published:** 2021-09-17

**Authors:** Kazunobu Sugihara, Masaki Tanito

**Affiliations:** Department of Ophthalmology, Shimane University Faculty of Medicine, Izumo 693-8501, Japan; ksugi@med.shimane-u.ac.jp

**Keywords:** age, central corneal thickness, Goldmann Applanation tonometer, non-contact tonometer, rebound tonometer, iCare

## Abstract

This study aimed to compare intraocular pressures (IOP) using different tonometers, Goldmann applanation (IOP_GAT_), non-contact (IOP_NCT_), and rebound (IOP_RBT_), and to assess the effects of aging and central corneal thickness (CCT) on the measurements. The IOP_GAT_, IOP_NCT_, IOP_RBT_, mean patient age (65.1 ± 16.2 years), and CCT (521.7 ± 39.2 µm) were collected retrospectively from 1054 eyes. The differences among IOPs were compared by the paired *t*-test. Possible correlations between devices, age, and CCT were assessed by linear regression analyses. The effects of age and CCT on the IOP reading were assessed by mixed-effects regression models. The IOP_GAT_ values were 2.4 and 1.4 mmHg higher than IOP_NCT_ and IOP_RBT_, respectively; the IOP_NCT_ was 1.0 mmHg lower than IOP_RBT_ (*p* < 0.0001 for all comparisons). The IOPs measured by each tonometer were highly correlated with each other (*r* = 0.81–0.90, *t* = 45.2–65.5). The linear regression analyses showed that age was negatively correlated with IOP_NCT_ (*r* = −0.12, *t* = −4.0) and IOP_RBT_ (*r* = −0.14, *t* = −4.5) but not IOP_GAT_ (*r* = 0.00, *t* = −0.2); the CCT was positively correlated with IOP_GAT_ (*r* = 0.13, *t* = 4.3), IOP_NCT_ (*r* = 0.29, *t* = 9.8), and IOP_RBT_ (*r* = 0.22, *t* = 7.2). The mixed-effect regression models showed significant negative correlations between age and IOP_NCT_ (*t* = −2.6) and IOP_RBT_ (*t* = −3.4), no correlation between age and IOP_GAT_ (*t* = 0.2), and a significant positive correlation between CCT and the tonometers (*t* = 3.4–7.3). No differences between IOP_GAT_ and IOP_RBT_ were seen at the age of 38.8 years. CCT affects IOPs from all tonometers; age affects IOP_NCT_ and IOP_RBT_ in different degrees. IOP_RBT_ tended to be higher than IOP_GAT_ in young subjects, but this stabilized in middle age and became higher in older subjects.

## 1. Introduction

Intraocular pressure (IOP) is the only known modifiable risk factor relevant to the treatment of glaucoma. Goldmann applanation tonometry (GAT) has been considered the “gold standard” for IOP measurement, although its readings are affected by central corneal thickness (CCT), corneal curvature, the modulus of corneal elasticity, and tear film [1]. Noncontact tonometry (NCT) using air-puff pressure has several favorable characteristics, including no corneal contact and no requirement for local anesthesia, which facilitates convenient use [1]. Rebound tonometry (RBT) uses the impact rebound principle by launching a magnetized probe against the cornea using a solenoid; the speed of deceleration of probe is measured and converted into the IOP [2]. There is no need for an air puff, corneal anesthesia, and slit-lamp mounting, and the measurement skill enables affordable, quick, and repeated IOP measurements even in children and very old patients [3].

Previously, many studies have reported excellent correlations between IOP readings and GAT and NCT or RBT IOPs, although the IOP values themselves varied among the tonometers [4,5,6,7,8,9,10,11,12,13,14,15,16]. Most previous studies have assessed the CCT as a surrogate for explaining the measurement difference among tonometers [4,5,6,7,8,9,10,11,12,13,14,15,16]; however, other parameters that possibly affect IOP differences among tonometers have not been studied extensively.

During the routine use of the various tonometers in the clinic, we realized that RBT may yield higher IOP readings than GAT in young patients, while this scenario was reversed in older patients. To test our suspicion, we compared the IOP readings of GAT, NCT, and RBT and investigated the effects of age and CCT on the IOP readings in subjects who visited our glaucoma clinic.

## 2. Subjects and Methods

### 2.1. Subjects

This retrospective study adhered to the tenets of the Declaration of Helsinki; the institutional review board (IRB) of Shimane University Hospital reviewed and approved the research. Based on the approval, written informed consent from each subject was waived; instead, the study protocol was posted at the study institutions to notify participants about the study. Among the 716 subjects who visited the glaucoma clinic of one author (MT) during April 2018 and March 2019, a review of the medical charts identified 1054 eyes of 544 subjects that fulfilled the inclusion criteria and were included in the analyses. The inclusion criteria were the measurement of IOPs using GAT (IOP_GAT_), NCT (IOP_NCT_), and RBT (IOP_RBT_) on the same day and the recording of the CCT. In our glaucoma clinic, the IOPs obtained using the three different devices and CCT were recorded as routine examinations during the initial patient visit; most data collected were obtained at the initial visit; however, when multiple records of a subject were eligible, the most recent data were collected. No exclusion criterion was set in this real-world data analysis study; accordingly, all subjects who met the inclusion criteria were consecutively included irrespective of glaucoma or non-glaucoma, newly diagnosed or follow-up patients, treated or untreated, and the presence or absence of corneal and other eye diseases. Typically, one glaucoma specialist (MT) used the GAT and RBT (iCARE Rebound Tonometer TA01i, M.E. Technica, Tokyo, Japan) to record the IOPs. One of nine certified orthoptists in our department recorded the IOP using a non-contact air-puff tonometer (TonoRef III, Nidek, Aichi, Japan), and the CCT was recorded using a corneal pachymeter equipped in a specular microscope (EM-3000, Tomey, Nagoya, Japan). No pre-planned calibration of the tonometers was performed for this study.

### 2.2. Statistical Analysis

The differences among the IOPs assessed using the three tonometers were compared using the paired *t*-test. Possible correlations between three devices, their differences, i.e., NCT minus GAT (IOP_NCT-GAT_), RBT minus GAT (IOP_RBT-GAT_), and RBT minus NCT (IOP_RBT-NCT_), age, and CCT were assessed by linear regression analyses. The effects of age and CCT on each tonometer were further assessed using a mixed-effects regression model in which each patient’s identification number was regarded as a random effect, and both age and CCT were regarded as fixed effects. All continuous data were expressed as the mean ± standard deviation (SD). All statistical analyses were performed using the JMP version 11.0 statistical software (SAS Institute, Inc., Cary, NC). *p* < 0.05 was considered statistically significant.

## 3. Results

The subject ages, CCTs, and IOPs measured using the different tonometers are summarized in Table 1. The IOP_GAT_ was 2.4 and 1.4 mmHg higher than the IOP_NCT_ and IOP_RBT_, respectively, and the IOP_NCT_ was 1.0 mmHg lower than IOP_RBT_ value (Table 1 and Figure S1A–C).

The IOPs measured by the different tonometers were highly correlated with each other (*r* = 0.81–0.90, *t* = 45.2–65.5) (Table 2 and Figure S2A–C). The linear regression analyses showed that the subjects’ ages were negatively correlated with the IOP_NCT_ (*r* = −0.12, *t* = −4.0) and the IOP_RBT_ (*r* = −0.14, *t* = −4.5) but not with the IOP_GAT_ (*r* = 0.00, *t* = −0.2) (Table 2 and Figure S3A–F). Age was also negatively correlated with the CCT (*r* = −0.13, *t* = −4.2) (Table 2 and Figure S4). The linear regression analyses showed that the CCT was positively correlated with the IOP_GAT_ (*r* = 0.13, 4.3), IOP_NCT_ (*r* = 0.29, *t* = 9.8), and IOP_RBT_ (*r* = 0.22, *t* = 7.2) (Table 2 and Figure S5A–F).

Finally, the effects of age and CCT on the IOPs measured by the three tonometers were assessed by mixed-effects regression models to adjust the interaction between age and CCT and cancel the bias resulting from the inclusion of both eyes of a subject (Table 3). Significant negative correlations were also seen between age and the IOP_NCT_ (*t* = −2.6) and IOP_RBT_ (*t* = −3.4), a non-significant correlation between age and the IOP_GAT_ (*t* = 0.2), and significant positive correlations between the CCT and all three tonometers (*t* = 3.4–7.3) (Table 3).

## 4. Discussion

As reported previously [4,5,6,7,8,9,10,11,12,13,14,15,16], the IOPs measured using the three devices were correlated with each other, and all were affected by the CCT (Table 2). A significant positive association between the CCT and IOP_NCT-GAT_ and IOP_RBT-GAT_ (Table 2) suggested a larger effect of the CCT on the IOPs obtained with NCT or RBT than with GAT, as reported previously [6,15].

**Figure 1:**
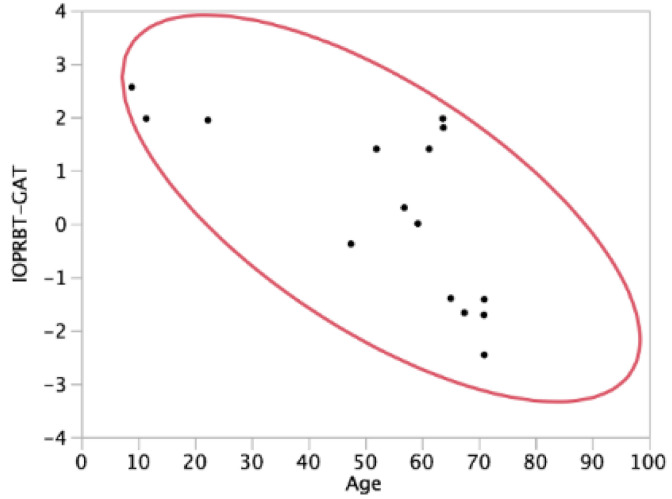
Correlations between subjects’ age (years) and intraocular pressure (IOP) (rebound tonometry minus Goldmann applanation tonometry) (RBT-GAT) (mmHg) in the current and previous studies. The scatterplots and a 90% bivariate normal ellipse are shown.

We identified a significant negative correlation between age and IOP_NCT_ or IOP_RBT_, while the correlation between age and IOP_GAT_ was not significant (Table 2). Since the IOP_RBT-NCT_ was negatively correlated with age (Table 2), the impact of age is the greatest on the RBT among the tonometers tested. The absolute *t*-value was the largest for age with the IOP_RBT-GAT_ (*t* = −7.6) among the models that included CCT and age (Table 3), suggesting that age determines the difference in IOP readings between GAT and RBT more than CCT. A recent report has found a negative correlation between IOP_RBT_ and age [17], and thus our results are in agreement with the previous report. Subject age and the detected difference between GAT and RBT readings in previous and current studies are summarized in Table 4. Including the current study, some studies have reported minus IOP_RBT-GAT_ values [10,12,13,14], while others have reported plus IOP_RBT-GAT_ values [4,5,6,7,8,11,15,16]; this discrepancy is not fully explained by the difference in the CCT. Scatterplots of the subjects’ ages and IOP_RBT-GAT_ from previous studies (Table 4and Figure 1) clearly suggest the roles of age and IOP_RBT-GAT_. Previously, 0 IOP_RBT-GAT_ was reported in subjects with a mean age of 59.3 years [9]. In the current subjects, based on linear regression analyses (Figure S3E), the age of subjects with 0 IOP_RBT-GAT_ was calculated to be 38.8 years. Thus, a lower/higher association of IOP readings between GAT and RBT is reversed based on the ages of the subjects. Other than the CCT, it has been proposed that corneal biomechanical properties such as corneal hysteresis (CH) and corneal resistance factors (CRF) affect the RBT and GAT differently [7,9,13]; both the CH and CRF decreased with aging [18]; thus, age-dependent changes in corneal biomechanical properties may be associated with our observation but need to be confirmed.

The limitations of the current study included the retrospective design and the inclusion of eyes with various types of glaucoma and glaucoma suspects. Because of the retrospective nature of the study, the methods of tonometry and examiners were not predetermined, although one examiner recorded the GAT and RBT using specific devices. We reviewed all patients who visited during the indicated period and included all patients who fulfilled the inclusion criteria, thus minimizing the selection bias. The inclusion of both eyes of a patient may have introduced bias, although we minimized this by using a mixed-effects regression model. Other than age and CCT, the modules of corneal elasticity [1] should affect the results. When the IOP elevates, the deviation between IOP_NCT_ and IOP_GAT_ becomes larger (Figure S2A); this may be explained by the effect of changes in corneal elasticity. Despite the various backgrounds of subjects included and the retrospective study design, we believe that our real-world data analysis is reasonable to test our suspicion, described in the introduction section.

## 5. Conclusions

In conclusion, CCT affects the IOP readings of GAT, NCT, and RBT, while age affects the NCT and RBT by different degrees. The RBT readings tended to be higher than the GAT readings in young subjects, but this stabilized in middle age and was reversed in older subjects.

## Figures and Tables

**Table 1:** Age, central corneal thickness (CCT), and intraocular pressures (IOPs) from 1054 eyes of 544 subjects.

Parameters	Mean ± SD	Range	Lower 95% CI	Upper 95% CI	*p* Value
Age, years	65.1 ± 16.2	11–96	63.7	66.4	-
CCT, µm	521.7 ± 39.2	337–675	519.4	524.1	-
IOP_GAT_, mmHg	16.9 ± 6.9	2–59	16.4	17.3	-
IOP_NCT_, mmHg	14.4 ± 5.5	2–47	14.1	14.7	-
IOP_RBT_, mmHg	15.4 ± 6.7	2–53	15.0	15.8	-
Differences in IOP between tonometers					
IOP_NCT-GAT_, mmHg	−2.4 ± 4.0	−41–+22	−2.7	−2.2	<0.0001
IOP_RBT-GAT_, mmHg	−1.4 ± 3.1	−15–+22	−1.2	−1.6	<0.0001
IOP_RBT-NCT_, mmHg	1.0 ± 3.4	−12–+26	0.8	1.2	<0.0001

The *p* values are calculated by using a paired *t*-test between each pair of tonometer groups. SD = standard deviation; CI = confidence interval.

**Table 2:** Possible correlations among age, CCT, and IOPs measured by each tonometer.

Parameters	Slope	Lower 95% CI	Upper 95% CI	*r*	*t*-Value	*p* Value
Correlation between tonometers (per mmHg)						
IOP_NCT_: IOP_GAT_	0.64	0.62	0.68	0.81	45.2	<0.0001
IOP_RBT_: IOP_GAT_	0.87	0.85	0.90	0.90	65.5	<0.0001
IOP_RBT_: IOP_NCT_	1.05	1.01	1.08	0.86	54.4	<0.0001
Correlation with age (per year)						
IOP_GAT_, mmHg	0.00	−0.03	0.02	0.00	−0.2	0.8736
IOP_NCT_, mmHg	−0.04	−0.06	−0.02	−0.12	−4.0	<0.0001
IOP_RBT_, mmHg	−0.06	−0.08	−0.03	−0.14	−4.5	<0.0001
IOP_NCT-GAT_, mmHg	−0.04	−0.05	−0.02	−0.16	−5.2	<0.0001
IOP_RBT-GAT_, mmHg	−0.05	−0.07	−0.04	−0.29	−9.7	<0.0001
IOP_RBT-NCT_, mmHg	−0.02	−0.03	0.00	−0.07	−2.4	0.0152
CCT, µm	−0.31	−0.45	−0.02	−0.13	−4.2	<0.0001
Correlation with CCT (per µm)						
IOP_GAT_, mmHg	0.02	0.01	0.03	0.13	4.3	<0.0001
IOP_NCT_, mmHg	0.04	0.03	0.05	0.29	9.8	<0.0001
IOP_RBT_, mmHg	0.04	0.03	0.05	0.22	7.2	<0.0001
IOP_NCT-GAT_, mmHg	0.02	0.01	0.02	0.17	5.6	<0.0001
IOP_RBT-GAT_, mmHg	0.01	0.01	0.02	0.18	5.8	<0.0001
IOP_RBT-NCT_, mmHg	0.00	−0.01	0.00	−0.04	−1.3	0.1961

The *t* and *p* values are calculated by linear regression analyses between each pair of indicated parameters. CI = confidence interval; *r* = Pearson’s correlation coefficient.

**Table 3:** Effects of age and CCT on the IOPs measured by each tonometer.

Parameters	Slope	Lower 95% CI	Upper 95% CI	*t*-Value	*p* Value
IOP_GAT_, mmHg					
Age (per year)	0.00	−0.03	0.03	0.2	0.8109
CCT (per µm)	0.02	0.01	0.03	3.4	0.0008
IOP_NCT_, mmHg					
Age (per year)	−0.03	−0.05	−0.01	−2.6	0.0088
CCT (per µm)	0.01	0.01	0.02	3.9	<0.0001
IOP_RBT_, mmHg					
Age (per year)	−0.05	−0.08	−0.02	−3.4	0.0008
CCT (per µm)	0.03	0.02	0.04	5.2	<0.0001
IOP_NCT-GAT_, mmHg					
Age (per year)	−0.03	−0.05	−0.02	−3.8	0.0002
CCT (per µm)	0.01	0.01	0.02	3.9	<0.0001
IOP_RBT-GAT_, mmHg					
Age (per year)	−0.05	−0.07	−0.04	−7.6	<0.0001
CCT (per µm)	0.01	0.00	0.01	3.3	0.0010
IOP_RBT-NCT_, mmHg					
Age (per year)	−0.02	−0.03	0.00	−2.3	0.0229
CCT (per µm)	0.00	−0.01	0.00	−1.4	0.1520

The *t* and *p* values are calculated by mixed-effect regression models to adjust the interaction between age and CCT and cancel the bias resulting from the inclusion of both eyes of a subject. CI = confidence interval; *r* = Pearson’s correlation coefficient.

**Table 4:** Summary of subjects’ age and IOP_RBT-GAT_ in previous studies.

Icare Model	Age, Years	IOP_RBT-GAT_, mmHg	Reference
iCareTa01i	63.8 ± 15.6	1.8 ± 2.8	4
iCareTa01i	61.3 ± 14.4	1.4 ± 2.7	5
iCareTa01i	52.0 ± 20.0	1.40 ± 2.19	6
iCareTa01i	22.3 ± 3.3	1.94 ± 2.75	7
iCarePro	63.7 ± 14.1	1.97 ± 3.29	8
iCarePro	59.3 ± 19.9	0.0	9
iCarePro	47.5 ± 105	−0.38	10
iCarePro	8.89 ± 3.41	2.56 ± 4.62	11
iCareTa01i	71.0 ± 7.5	−2.46 ± 2.10	12
iCarePro	71.0 ± 7.5	−1.42 ± 2.35	12
iCareTa01i	67.5 ± 10.9	−1.67 ± 3.07	13
iCareTa01i	70.95 ± 7.76	−1.71	14
iCarePro	56.9 ± 18.3	0.3	15
iCarePro	11.44 ± 2.31	1.97 ± 0.15	16
iCareTa01i	65.1 ± 16.2	−1.4 ± 3.1	Current study

The data are expressed as mean ± standard deviation.

## Data Availability

The data are fully available upon reasonable request to the corresponding author.

## Supplementary Materials

The following are available online at https://www.mdpi.com/article/10.3390/jcm10184202/s1, Figure S1: Differences in the intraocular pressure (IOP) (mmHg) among the tonometers, Figure S2: Correlations between the intraocular pressure (IOP) (mmHg) measured using different tonometers, Figure S3: Correlations between age (years) and intraocular pressure (IOP) (mmHg) measured using different tonometers, Figure S4: Correlations between age (years) and central corneal thickness (CCT) (µm), Figure S5: Correlations between the central corneal thickness (CCT) (µm) and intraocular pressure (IOP) (mmHg) measured using different tonometers.
