# Supplementary material for: Different Effects of Aging on Intraocular Pressures Measured by Three Different Tonometers
Source: J Clin Med. 2021 Sep 17;10(18):4202. doi: 10.3390/jcm10184202 (PMC8471438; doi:10.3390/jcm10184202)
Supplement: Supplementary file 1 [file jcm-10-04202-s001.zip › jcm-1382555-supplementary.pdf]

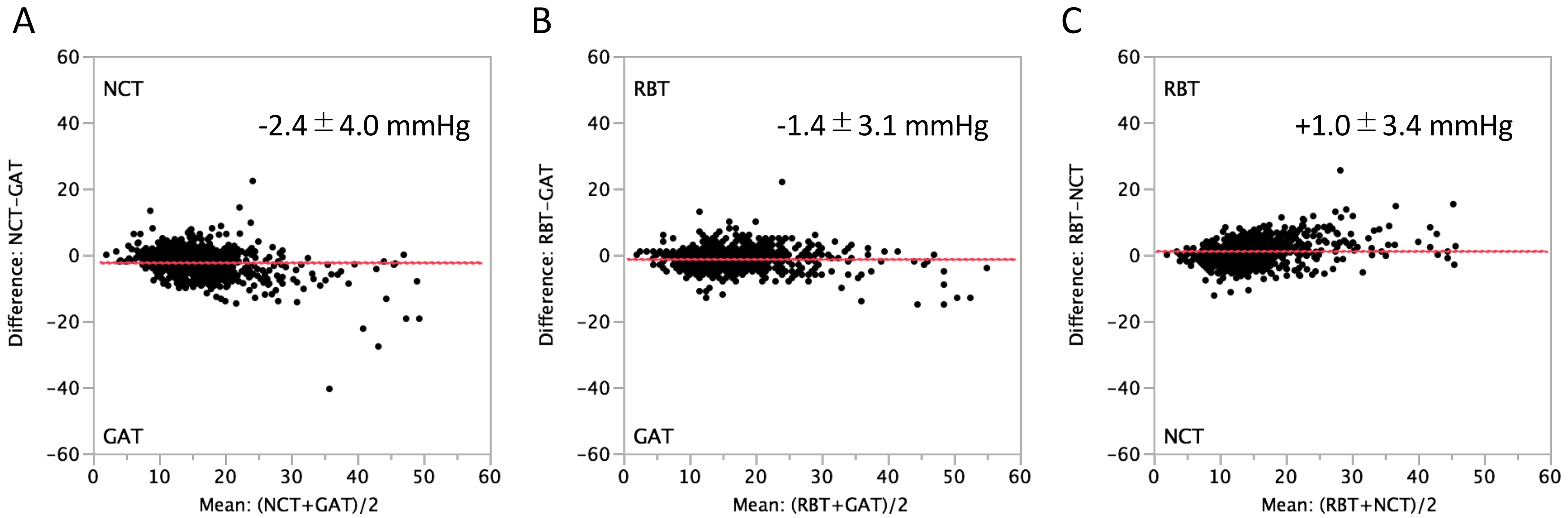

Supplementary FIGURE S1. Differences in the intraocular pressure (IOP) (mmHg) among the tonometers. The Bland-Altman plots show comparisons of noncontact tonometry (NCT) and Goldmann applanation tonometry (GAT) (A), rebound tonometry (RBT) and GAT (B), and RBT and NCT (C). The red lines indicate the mean differences in IOP.

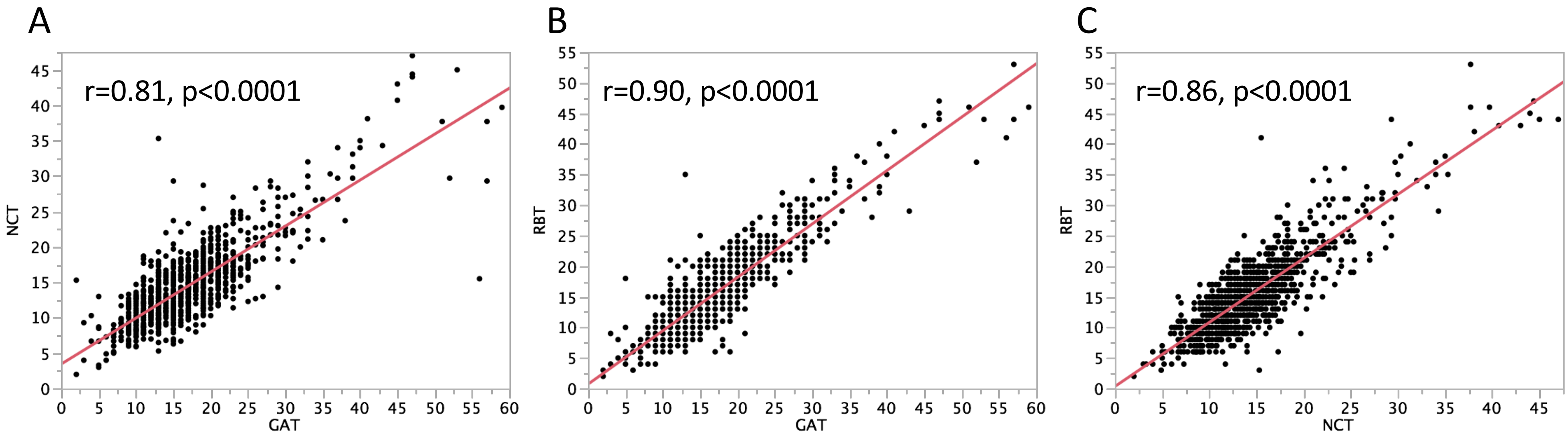

Supplementary FIGURE S2. Correlations between the intraocular pressure (IOP) (mmHg) measured using different tonometers.

The scatterplots and regression lines show comparisons of noncontact tonometry (NCT) and Goldmann applanation tonometry (GAT) (A), rebound tonometry (RBT) and GAT (B), and RBT and NCT (C). r indicates Pearson’s correlation coefficient.

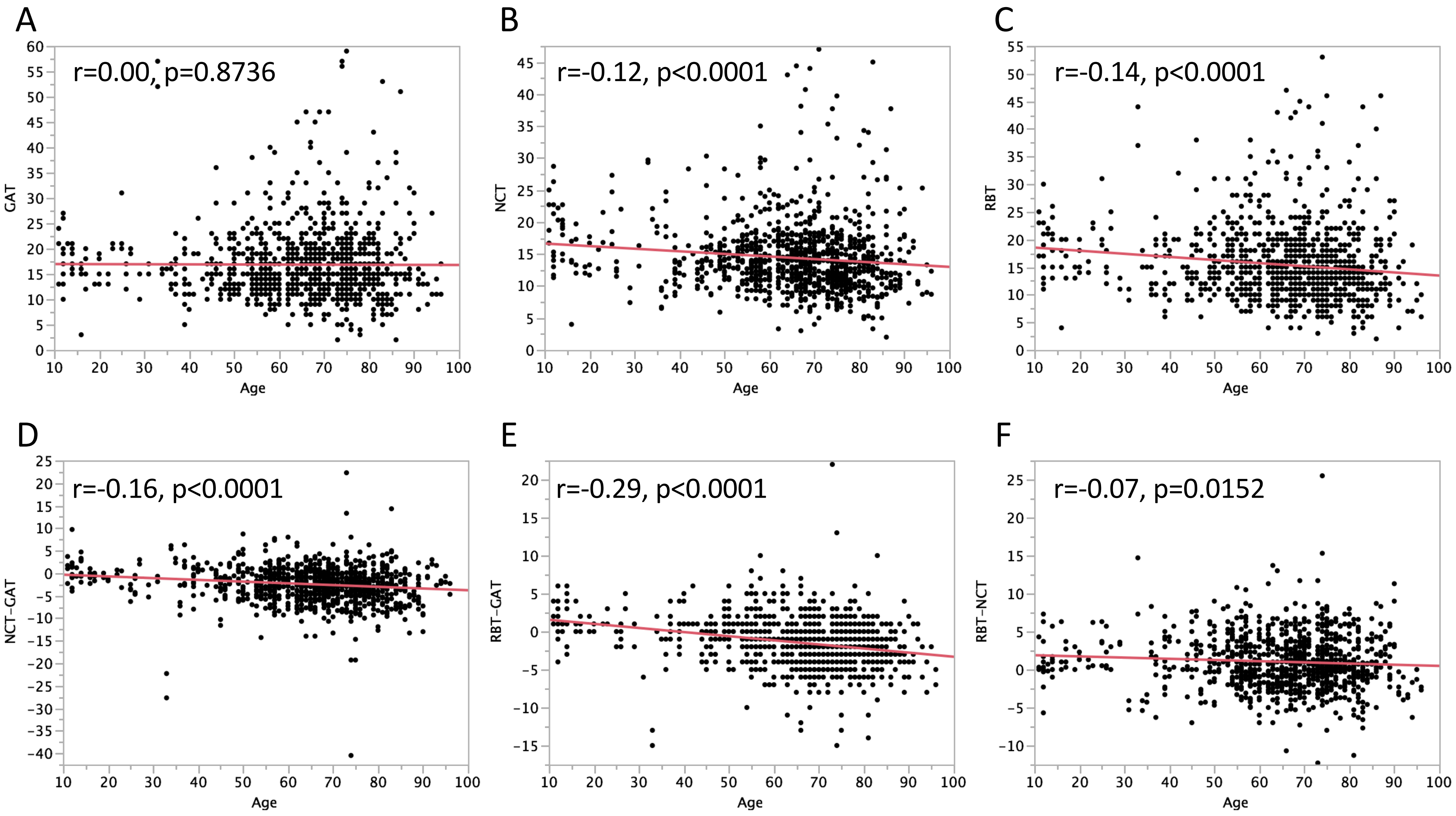

Supplementary FIGURE S3. Correlations between age (years) and intraocular pressure (IOP) (mmHg) measured using different tonometers.

The scatterplots and regression lines show comparisons of age and Goldmann applanation tonometry (GAT) (A), noncontact tonometry (NCT) (B), rebound tonometry (RBT) (C), NCT-GAT (D), RBT-GAT (E), and RBT-NCT (F). r indicates Pearson's correlation coefficient.

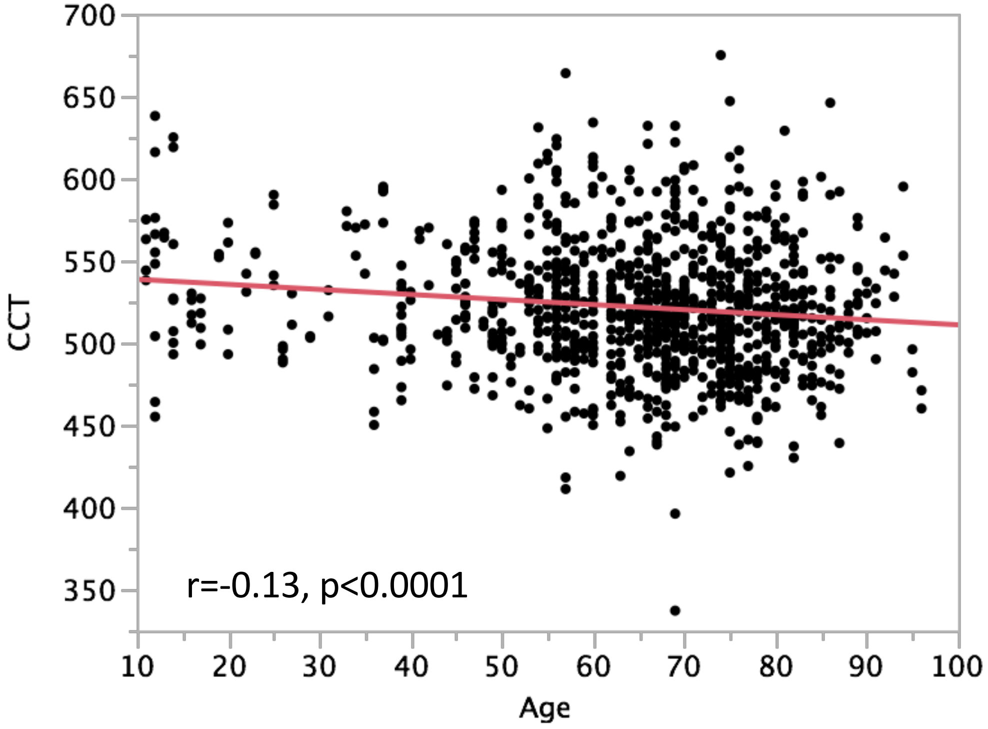

Supplementary FIGURE S4. Correlations between age (years) and central corneal thickness (CCT) (μm).

The scatterplots and regression line show comparisons of age and central corneal thickness (CCT). r indicates Pearson's correlation coefficient.

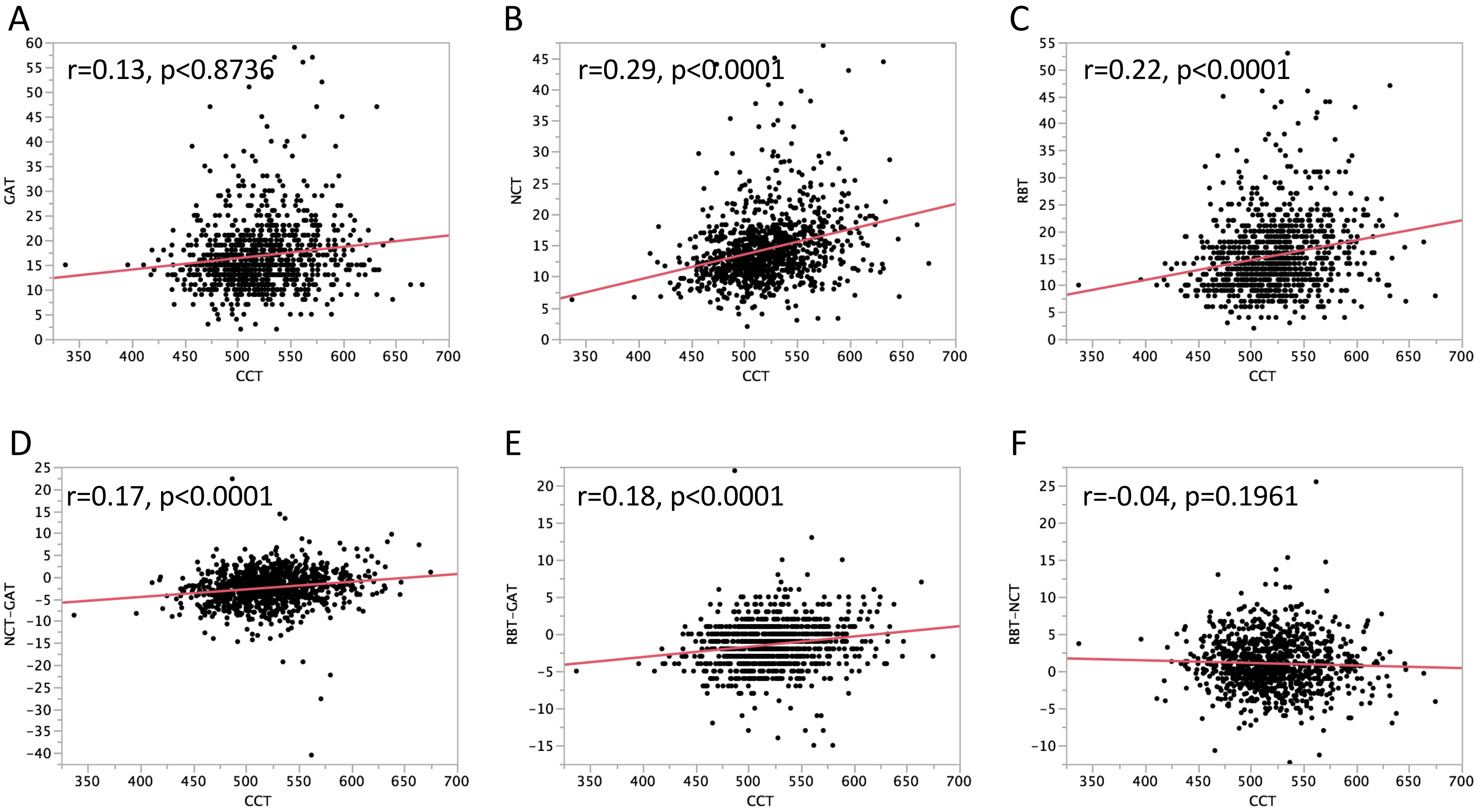

Supplementary FIGURE S5. Correlations between the central corneal thickness (CCT) (μm) and intraocular pressure (IOP) (mmHg) measured using different tonometers.
The scatterplots and regression lines show comparisons of the central corneal thickness (CCT) and Goldmann applanation tonometry (GAT) (A), noncontact tonometry (NCT) (B), rebound tonometry (RBT) (C), NCT-GAT (D), RBT-GAT (E), and RBT-NCT (F). r indicates Pearson's correlation coefficient.
